# Plant natural products targeting NLRP3 inflammasome in Parkinson's disease: Molecular activation and regulation to therapeutics

**DOI:** 10.1016/j.isci.2026.115257

**Published:** 2026-03-06

**Authors:** Hao Lu, Fengyi Zeng, Jiao Sun, Baiyang Li, Zhimei Lei, Chenyu Zeng, Chendan Zhu, Yanmei Sheng, Quekun Peng, Yiran Sun, Shiyan Zhang

**Affiliations:** 1 Department of Scientific Research Management, Zhoukou Central Hospital, Zhoukou, Henan 466000, P.R. China; 2 Key Laboratory of Structure-Specific Small Molecule Drugs at Chengdu Medical College of Sichuan Province, School of Pharmacy, Chengdu Medical College, Chengdu 610500, China; 3 School of Biosciences and Technology, Sichuan Higher Education Institute Key Laboratory of Major Disease Target Discovery and Protein Drug Development, Chengdu Medical College, Chengdu 610500, Sichuan Province, China; 4 The First Affiliated Hospital of Traditional Chinese Medicine of Chengdu Medical College·Xindu Hospital of Traditional Chinese Medicine, Chengdu Medical College, Chengdu 610500, Sichuan Province, China

**Keywords:** health sciences, medicine, natural product chemistry, neurology

## Abstract

Parkinson disease (PD) is a progressive neurodegenerative disorder characterized by the loss of dopaminergic neurons and the pathological accumulation of α-synuclein (α-syn), a key neuronal protein implicated in neuroinflammation and disease progression. The NOD-like receptor protein 3 (NLRP3) inflammasome, a critical component of the innate immune system, serves as a macromolecular sensor for damage-associated molecular patterns (DAMPs) and pathogen-associated molecular patterns (PAMPs). Its aberrant activation drives chronic neuroinflammation, which exacerbates PD pathology. This review elucidates the molecular mechanisms underlying NLRP3 inflammasome activation and its intricate relationship with PD, emphasizing the role of α-syn as a DAMP that triggers NLRP3 via Toll-like receptors (TLRs) and mitochondrial dysfunction. The review highlights how mitochondrial impairment and lysosomal disruption amplify NLRP3 activation, creating a vicious cycle of neuroinflammation and neuronal death. Importantly, emerging evidence demonstrates that plant natural products (PNPs) can effectively target the NLRP3 inflammasome pathway, offering a promising avenue for PD therapy. This review not only establishes the NLRP3 inflammasome as a pivotal macromolecular target in PD therapy but also systematically demonstrates, from the perspectives of structural biology, immunology, and translational medicine, the significant value of PNPs as a novel class of precision neuroprotective modulators—thereby laying a theoretical foundation for developing multi-target, low-toxicity therapeutic agents against PD.

## Graphical abstract

**Figure:**
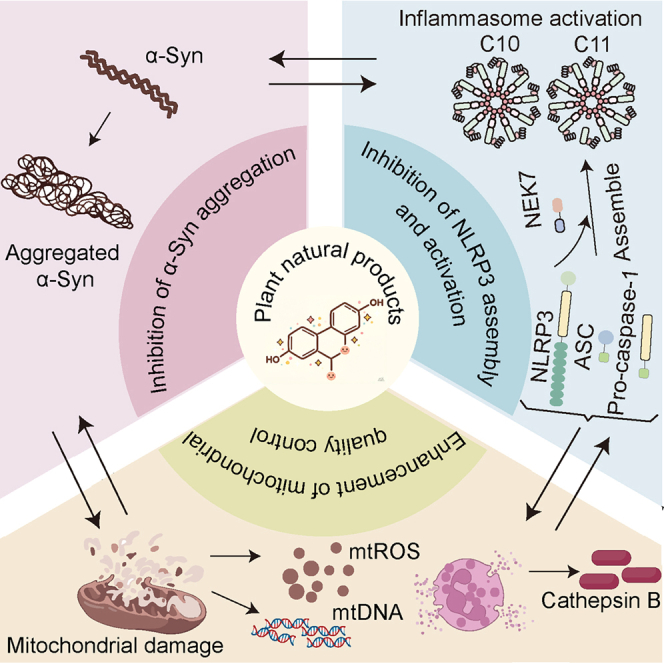

## Introduction

Parkinson disease (PD), characterized by the loss of dopaminergic (DA) neurons in the substantia nigra compacta (SNpC),^1^ is a chronic progressive neurodegenerative disorder associated with aging. Chronic neuroinflammation triggered by the intra-neuronal accumulation of pathologic α-synuclein (α-syn) is a major pathological hallmark of PD.^2^ The number of PD patients worldwide is expected to increase by more than 50% by 2030.^3^ PD has been recognized as the leading cause of disability in the elderly.^4^ The current treatment for PD primarily consists of pharmacological approaches, including levodopa-based agents,^5^ dopamine agonists,^6^ and anti-cholinergics.^7^ Novel therapies, such as immunotherapy,^8^ stem cell therapy,^9^ and nanomaterial-based therapies,^10^ are still in the exploratory stage, with many challenges needing to be addressed. Given the increasing number of patients and the financial burden and physical harm caused by long-term medication, there is an urgent need to identify new targets or mechanisms of action, which are crucial for developing PD drugs.

Neuroinflammation serves as a vital defense mechanism in the central nervous system (CNS) and plays a crucial role in the pathogenesis of PD.^11^^,^^12^ An increasing number of studies suggest that acute short-term activation of immune cells can protect the nervous system,^13^ repair damaged tissues, and eliminate toxins and pathogens.^14^^,^^15^^,^^16^ Acute inflammation is generally favorable, whereas chronic inflammation is associated with neurodegenerative diseases.^17^ Prolonged inflammation has the potential to disrupt neurochemical processes of the CNS, potentially causing neuronal death, defects in cellular maintenance, disruption of the blood-brain barrier (BBB), and, ultimately, an overactive inflammatory response.^18^ Inflammatory complexes, represented by NOD-like receptor protein 1 (NLRP1),^19^ NOD-like receptor protein 3 (NLRP3),^20^ and Absent in Melanoma 2 inflammasome 2 (AIM2),^21^ are involved in innate immunity, and their abnormal activation contributes to the development of various inflammation-related human disorders. Among various inflammatory signaling pathways, the NLRP3 inflammasome has emerged as a key molecular hub that transduces multiple pathogenic signals, including those derived from α-syn aggregates, into robust inflammatory responses. Its activation is particularly relevant in the transition from acute, protective neuroinflammation to the chronic, detrimental state observed in PD progression. This suggests a close association between NLRP3 inflammatory complexes and PD pathology.

Research has demonstrated that NLRP3-knockout 1-methyl-4-phenyl-1,2,3,6-tetrahydropyridine (MPTP) mouse model exhibits significantly reduced neuroinflammation and protection of DA neurons,^22^ indicating that NLRP3 is a key component in the initiation of neuroinflammation. Complementing these preclinical findings, clinical studies report elevated levels of NLRP3 component in the serum and cerebrospinal fluid of PD patients,^23^ underscoring its critical role in PD pathology. Furthermore, caspase-1 is closely linked to PD pathology, as it participates in cleaving α-syn and promoting its aggregation.^24^ MPTP model mice lacking caspase-1 show attenuated PD pathology and increased resistance to substantia nigra neuronal damage.^25^ These lines of evidence collectively establish the NLRP3 inflammasome as a key driver of PD-associated neuroinflammation.

The NLRP3 inflammasome, a crucial component of the innate immune system, is composed of NLRP3, apoptosis-associated speck-like protein containing a CARD (ASC), and NIMA-related kinase 7 (NEK7), along with the pro-caspase-1 protein.^26^^,^^27^^,^^28^ Aggregated α-syn acts as a damage-associated molecular pattern (DAMP) and further activates the NLRP3 inflammasome, exacerbating neuroinflammation in PD and resulting in behavioral abnormalities.^29^^,^^30^^,^^31^ Furthermore, activation of the NLRP3 inflammasome triggers the release of inflammatory factors that promote the aggregation of α-syn, contributing to the pathogenesis of PD.^30^ Studies have demonstrated that the application of the NLRP3-specific inhibitor compound MCC950 can alleviate PD pathology.^32^^,^^33^ Nevertheless, targeting NLRP3 for PD therapy is confronted with several key challenges: debate persists over upstream activating signals such as mitochondrial reactive oxygen species (mtROS); the mechanisms of most multi-targeting plant natural products (PNPs) remain unclear and are hampered by poor BBB permeability and limited clinical evidence; and the cell type-specific functions of NLRP3 in the CNS require further elucidation to enable precise therapeutic interventions.

Therefore, systematically elucidating the multi-faceted regulatory network of NLRP3 in PD, while critically evaluating both the advantages and current bottlenecks of intervention strategies represented by PNPs, is crucial for defining future research directions and advancing NLRP3-targeted neuroprotective therapies from concept to clinic.

To ensure a comprehensive and up-to-date analysis, the literature cited in this review was systematically retrieved from major scientific databases, including PubMed, Web of Science, and Scopus, covering publications up to January 2026. The search strategy employed a combination of keywords and MeSH terms related to “Parkinson’s disease,” “NLRP3 inflammasome,” “neuroinflammation,” “α-synuclein,” and “plant natural products” or “molecular.” Primary inclusion criteria were: (1) original research articles, reviews, and meta-analyses published in English; (2) studies focusing on the molecular mechanisms linking NLRP3 inflammasome activation to PD pathogenesis; and (3) preclinical or clinical investigations evaluating plant-derived compounds as NLRP3 modulators in PD models. Articles were excluded if they were not peer reviewed, lacked direct relevance to PD or NLRP3, or presented insufficient mechanistic detail. The final selection aimed to highlight key mechanistic insights and promising therapeutic candidates while reflecting the current state of the field.

## NLRP3 inflammasome

### The formation of NLRP3 inflammasome

Structurally, the NLRP3 inflammasome comprises a sensor (NLRP3), an adaptor (ASC), and an effector (pro-caspase-1).^28^^,^^34^ NLRP3 encompasses a pyrin domain (PYD) at its N terminus, a central nucleotide-binding oligomerization domain (NACHT), and a C-terminal leucine-rich repeat (LRR) domain. ASC features an N-terminal PYD and a C-terminal caspase-recruitment domain (CARD). Caspase-1 consists of three domains: an N-terminal CARD, a central large catalytic domain (p20), and a C-terminal small catalytic subunit domain (p10).^35^ Recent structural and functional studies have demonstrated that the mitotic kinase NEK7 mediates NLRP3 inflammasome activation by binding to the LRR domain of NLRP3.^36^^,^^37^ Disruption of this specific interaction, as revealed by recent structural insights, effectively impedes inflammasome assembly and activation, underscoring the critical role of the NEK7-NLRP3 interface in this process.^38^ As a crucial component of the innate immune system, NLRP3 remains autoinhibited under resting conditions via intramolecular interactions between its NACHT and LRR domains, which prevent its association with the adaptor protein ASC and thereby block inflammasome assembly.^39^ Upon acute or chronic inflammatory or stress stimulation, NLRP3 becomes activated and oligomerizes into a multi-protein complex.^40^^,^^41^

### Mechanism of NLRP3 inflammasome activation

**Figure 1:**
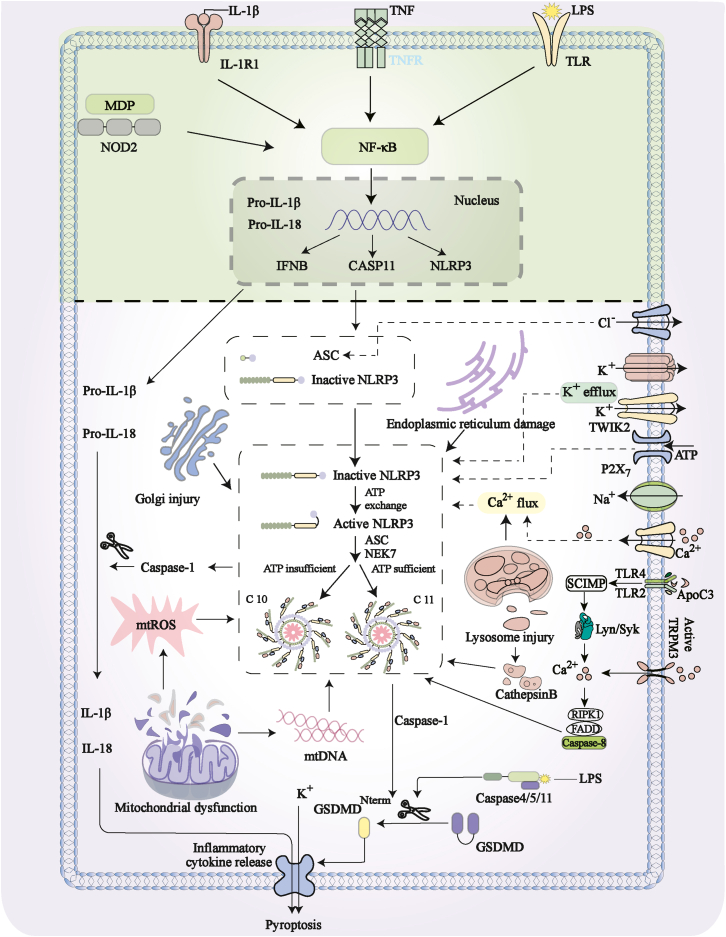
NLRP3 inflammasome activation and signaling pathways During the initiation phase, NF-κB translocates to the nucleus upon receiving signaling cues generated by the engagement of PAMPs, including LPS, and certain cytokines with their respective receptors, as well as by the recognition of MDP by the NOD2 receptor. This translocation facilitates the upregulation of inflammasome components and the expression of cytokines associated with both the classical and non-classical pathways. During the activation phase, signaling predominantly arises from ion flux events and organelle damage, inducing NLRP3 activation, followed by its recruitment of NEK7, ASC, and pro-caspase-1 to form the NLRP3 inflammasome. Notably, damage to the Golgi apparatus and endoplasmic reticulum directly triggers NLRP3 inflammasome assembly and activation, while mitochondrial and lysosomal damage, respectively, promote NLRP3 inflammasome assembly and activation through the release of mtROS, mtDNA, and cathepsin B. Subsequently, activated caspase-1 cleaves pro-IL-1β and pro-IL-18 into mature IL-1β and IL-18, respectively. Additionally, it collaborates with caspase-4/5/11 from the non-classical activation pathway induced by LPS, participating in the cleavage and activation of GSDMD. Notably, alternative pathways mediated by TLR2/4 and TRPM3 are also involved in the assembly and activation of NLRP3 inflammation. This results in the generation of its active N-terminal domain, which associates with lipid bilayers of cell membrane, inducing pore formation and consequent efflux of cellular contents including potassium ions and pro-inflammatory cytokines, thereby instigating inflammation and cell death. Created with Adobe Illustrator 2025. PAMPs, pathogen-associated molecular patterns; LPS, lipopolysaccharide; MDP, muramyl dipeptide; NOD2, nucleotide-binding oligomerization domain 2; NEK7, NIMA-related kinase 7; ASC, apoptosis-associated speck-like protein containing a CARD; GSDMD, gasdermin D; TLR, Toll-like receptor.

The activation of the NLRP3 inflammasome is driven by a wide range of endogenous and exogenous signals, including pathogens, cellular damage, and stress factors. Upon activation, the NLRP3 inflammasome stimulates the production and release of pro-inflammatory cytokines, particularly IL-1β and IL-18, which play a pivotal role in inflammatory responses. The activation of the NLRP3 inflammasome is tightly regulated by the host and can occur through distinct molecular pathways, broadly categorized into the canonical, non-canonical, and alternative pathways (Figure 1).

#### Classical activation pathway

The activation process of the NLRP3 inflammasome is tightly regulated by the host and involves two distinct phases termed “priming” and “activation.”^42^ The initiation phase triggers the transcription of genes associated with the NLRP3 inflammasome by providing an initial signal. Upon stimulation by pathogen-associated molecular patterns (PAMPs) or cytokines,^43^ such as Toll-like receptors (TLRs), interleukin 1 receptor 1 (IL-1R1), tumor necrosis factor receptor 1 (TNFR1), and nucleotide-binding oligomerization domain-containing protein 2 (NOD2), the nuclear factor-κB (NF-κB) signaling cascade is facilitated, upregulating the transcriptional expression of the intracellular inflammasome components NLRP3, pro-IL-1β, and pro-IL-18.^44^^,^^45^ The activation phase is orchestrated by various molecular and cellular effectors mediated by certain PAMPs and DAMPs, including K^+^ efflux,^46^ Ca^2+^ efflux,^47^ lysosomal disruption,^48^ and mtROS generation,^49^ with K^+^ efflux being recognized as a primary activation mechanism.^50^ Upon induction of NLRP3 activation, its N-terminal PYD interacts with the PYD of ASC, leading to ASC-mediated recruitment of the effector molecule pro-caspase-1 via the CARD, triggering the assembly of the NLRP3 inflammasome. Consequently, pro-caspase-1 undergoes self-cleavage, transitioning into its active form, caspase-1, which cleaves pro-IL-1β and pro-IL-18 into mature IL-1β and IL-18, respectively. Additionally, caspase-1 cleaves gasdermin D (GSDMD), a critical protein in pyroptosis, leading to cell rupture and release of more pro-inflammatory cytokines,^51^ further amplifying the inflammatory response.

#### Non-canonical activation pathway

The non-canonical signaling pathway is mediated by lipopolysaccharide (LPS). After Gram-negative bacteria are phagocytosed and degraded by immune cells, released LPS directly binds to and activates mouse caspase-11 or human caspase-4/5, promoting oligomerization and auto-processing of these caspases, which, in turn, triggers non-canonical activation of the NLRP3 inflammasome.^52^ These enzymes cleave GSDMD to generate GSDMD-N, which binds to cardiolipin, phosphatidylinositol, and phosphatidylserine in the plasma membrane to form pores, promoting K^+^ efflux and triggering pyroptosis.^53^ Furthermore, activation of caspase-4/5/11 induces pannexin-1 (PANX1) activation, driving ATP efflux. Extracellular ATP binding to the P2X7 receptor leads to K^+^ efflux and ion channel opening, thereby activating the NLRP3 inflammasome. Through a caspase-1-dependent mechanism, this process initiates pyroptosis, effectively converging the canonical pathway driven by caspase-1 and the non-canonical pathway governed by caspases-4/5/11.^54^ Thus, as a core executor of pyroptosis, the NLRP3 inflammasome not only amplifies the inflammatory cascade but also plays a key role in regulating immune homeostasis.

In the pathological context of PD, the non-canonical NLRP3 activation pathway may play a significant role via the “gut-brain axis” mechanism.^55^ PD patients often exhibit gut microbiota dysbiosis and intestinal barrier dysfunction, leading to the translocation of Gram-negative bacterial LPS into the systemic circulation.^56^ This circulating LPS can cross the BBB or travel via vagal nerve afferents, where it is sensed by immune cells within the CNS, such as microglia. Upon entering the cytosol, LPS can directly activate caspase-4/5/11, which subsequently cleaves GSDMD to form membrane pores. This event triggers a dual effect: it not only directly induces pyroptosis but also, through the formation of pores that facilitate K^+^ efflux, converges with and potently amplifies the canonical NLRP3-caspase-1 inflammatory pathway.^57^ Therefore, in PD, the non-canonical pathway does not operate in isolation. It likely acts as a critical bridge linking peripheral gut inflammation to central neuroinflammation, transforming systemic low-grade inflammatory signals into intensified inflammasome activation and neuronal damage within the brain. This provides a novel explanatory dimension for the origin and progression of neuroinflammation in PD.

#### Alternative activation pathway

In the alternative activation pathway of the NLRP3 inflammasome, TLR4 recognizes LPS and triggers the TRIF-RIPK1-FADD-CASP8 signaling axis, which directly activates the assembly of the NLRP3-ASC-pro-caspase-1 complex, thereby promoting the maturation and release of IL-1β/IL-18 independently of K^+^ efflux, ASC speck formation, or pyroptosis.^58^ Apolipoprotein C3 (ApoC3) further amplifies this pathway by forming a heterodimer with TLR2/TLR4, initiating the SCIMP-Lyn-Syk-TRPM2 cascade that leads to Ca^2+^ influx, ROS burst, and NADPH oxidase activation, ultimately resulting in the specific activation of caspase-8 and NLRP3 inflammasome.^59^ Additionally, defects in endocytic trafficking, such as lysosomal stress, can reposition NLRP3 to endolysosomes via phosphatidylinositol 4-phosphate (PI4P) anchoring, placing it in a “primed” state.^60^ Upon TLR activation or the release of DAMPs from damaged cells, NLRP3 becomes fully activated, triggering IL-1β release and pyroptosis, thereby driving chronic sterile inflammation.^61^ Considering that PD is a slowly progressive degenerative disease in which α-syn aggregation continuously stimulates microglia and sustains inflammatory activation, this pathway may play a critical role in PD pathology. However, its specific mechanisms still warrant further in-depth investigation.

In the neuroinflammatory progression of PD, the activation of the NLRP3 inflammasome follows a dynamic and competitive network model: multiple cellular stress signals cooperate or compete with each other to collectively determine its final activation state. Here, K^+^ efflux serves as a central triggering hub, providing a final common pathway for various upstream events. Mitochondrial and lysosomal damage, which are directly linked to α-syn pathology and defects in organelle autophagy, represent disease-specific triggers and amplifiers. Persistent metabolic dysregulation and oxidative stress, in turn, act as “priming” factors that continuously lower the activation threshold of NLRP3. In the chronic course of PD, these pathways do not proceed linearly but rather interweave and self-amplify: initial protein aggregation triggers acute inflammation, which, in turn, exacerbates organelle dysfunction and metabolic imbalance. The latter then primes glial cells to overreact to minor stimuli, thereby forming a self-perpetuating cycle of chronic neuroinflammation that drives progressive neuronal loss.

#### Other activation pathway

Recent studies reveal that biomolecular condensates, such as phase-separated liquid droplets, play a critical role in regulating NLRP3 inflammasome activation. For instance, zinc finger DHHC domain-containing protein 7 (ZDHHC7)-mediated palmitoylation of NLRP3 drives its phase separation, lowering the activation threshold in response to diverse stimuli like K^+^ efflux or palmitate.^62^ Similarly, stress-induced phase separation of p62 protein transforms autophagic droplets into stress granules, which recruit the NLRP3 adaptor ASC to assemble the inflammasome.^63^ In ischemic injury, small ubiquitin-like modifier 1 (SUMO1) upregulation enhances HIF-1α stability through nucleolin (Ncl)-mediated phase separation, promoting NLRP3 expression and pyroptosis.^64^ Additionally, YTHDF1 undergoes phase separation to form stress granules, driving circadian locomotor output cycles kaput (CLOCK) translation and amplifying NLRP3-driven airway inflammation.^65^ Counterintuitively, amphiphilic molecules like doxorubicin directly induce NLRP3 phase separation independent of palmitoylation, bypassing ZDHHC7-mediated regulation.^62^ These findings highlight how condensate dynamics fine-tune NLRP3 activation in response to cellular stress.

Long noncoding RNAs (lncRNAs) emerge as key regulators of NLRP3 inflammasome activity, functioning through diverse mechanisms. In lung cancer, LINC00969 suppresses NLRP3 expression via dual epigenetic modulation—histone H3K27 trimethylation and m6A methylation—promoting EGFR-TKI resistance.^66^ The lncRNA 4344/miR-138-5p/NLRP3 axis drives neuroinflammation and cognitive impairment by enhancing microglial pyroptosis.^67^ MIR181A1HG acts as a molecular decoy for Foxp1, activating NLRP3 in endothelial cells and exacerbating atherosclerosis.^68^ METTL14-mediated m6A modification of lncRNA TINCR stabilizes its mRNA, upregulating NLRP3 and promoting diabetic cardiomyopathy.^69^ In cerebral ischemia-reperfusion injury, Gm44206 knockdown alleviates microglial pyroptosis by inhibiting the NLRP3/caspase-1/GSDMD pathway.^70^ Platr4, a circadian-regulated lncRNA, suppresses NLRP3 transcription by blocking NF-κB binding to its promoter.^71^ Conversely, IncMCL1 inhibits neuronal pyroptosis in epilepsy by destabilizing DDX3X via ubiquitination.^72^ Retinal ischemia-reperfusion injury activates the 181-Rik/NLRP3 axis by disrupting mitochondrial dynamics and ROS production.^73^ Post-stroke pyroptosis is mediated by the Tug1/miR-145a-5p/Tlr4 axis, linking lncRNA dysregulation to neuroinflammation.^74^ In allergic rhinitis, NEAT1 scaffolds PTBP1 to stabilize FOXP1 mRNA, activating NLRP3-mediated epithelial pyroptosis.^75^ These studies underscore the complexity of lncRNA-mediated NLRP3 regulation across diseases.

Within the regulatory network of the NLRP3 inflammasome, a variety of post-translational modifications (PTMs) act synergistically during its critical “priming” and “activation” stages, constituting a sophisticated molecular switch system. Among them, ubiquitination and deubiquitination play a crucial role in the priming stage; for instance, K48-linked ubiquitination mediated by SCF-FBXL2 targets NLRP3 for degradation,^76^ whereas deubiquitinases like OTUD6A stabilize the protein by removing ubiquitin chains.^77^ Phosphorylation and dephosphorylation exert bidirectional regulatory functions during both priming and activation. For example, phosphorylation of the S5 site by AKT exerts an inhibitory effect,^78^^,^^79^ while tyrosine phosphorylation mediated by JNK1 and BTK promotes its oligomerization and activation.^80^^,^^81^^,^^82^ The palmitoylation modification, which has recently garnered significant attention, is catalyzed by ZDHHC enzymes.^83^ It drives the recruitment and anchoring of NLRP3 to membranous structures such as the Golgi apparatus, which is indispensable for its proper localization and activation.^84^

Additionally, acetylation exerts a bidirectional regulatory effect, wherein KAT5 promotes but SIRT2 inhibits NLRP3 activity.^85^^,^^86^ SUMOylation and deSUMOylation, mediated by enzymes such as TRIM28 and SENP6/7, respectively, are crucial for modulating the stability of NLRP3.^87^^,^^88^ Furthermore, several novel regulatory modifications have emerged as significant mechanisms, encompassing ISGylation that stabilizes NLRP3,^89^ the itaconate-induced alkylation of NLRP3 that disrupts the NLRP3-NEK7 interaction,^90^ and UFMylation that contributes to maintaining NLRP3 stability.^91^ Together, they form a complex and dynamic PTM regulatory network that precisely controls the activation threshold, timing, and intensity of the NLRP3 inflammasome.

The preceding discussion provides a general overview of the structural characteristics and molecular mechanisms underlying the activation of the NLRP3 inflammasome. In the following section, we turn our focus to the relationship between the NLRP3 inflammasome and the pathology of PD, elucidating the mechanistic rationale for suppressing PD progression by inhibiting NLRP3 inflammasome activation.

## Relationship between NLRP3 inflammasome and PD pathogenesis

### α-Syn regulates the activation of the NLRP3 inflammasome

**Figure 2:**
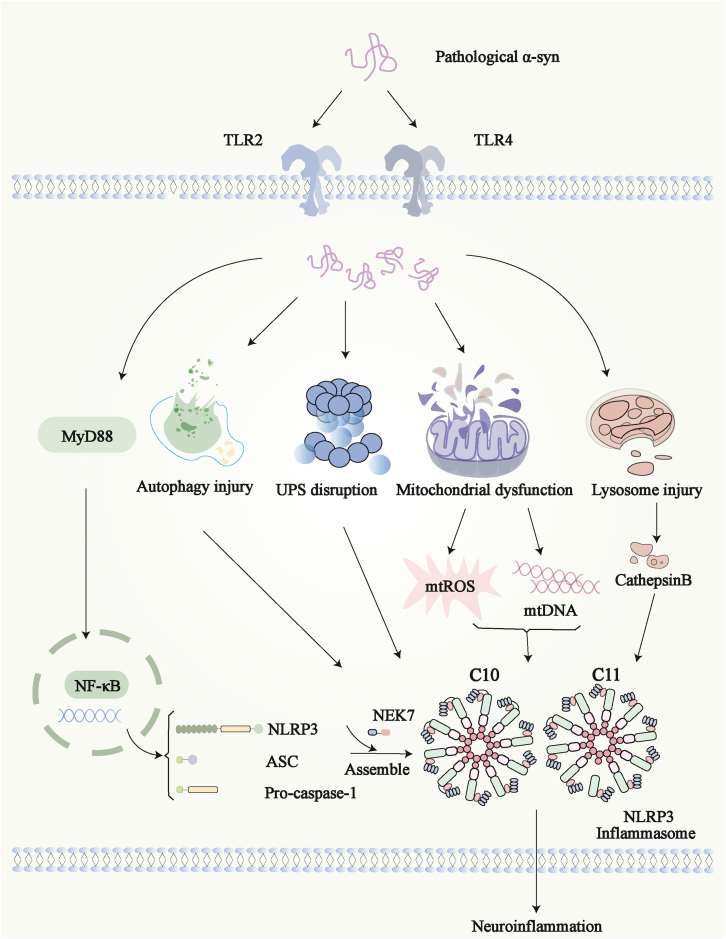
Schematic of NLRP3 inflammasome activation in PD pathogenesis **A**ctivation of the NF-kB signaling pathway promotes the formation of NLRP3, ASC, and pro-caspase-1 complexes, and these complexes then bind to NEK7 to activate the NLRP3 inflammasome. Furthermore, pathological α-syn not only promotes disintegration of autophagy and UPS but also impairs mitochondrial and lysosome to release abundant mtROS, mtDNA, and cathepsin B, respectively. It can also exert effects on NLRP3 to cause neuroinflammation. Created with Adobe Illustrator 2025. MyD88, recombinant myeloid differentiation factor 88; TLRs, toll-like receptors; mtDNA, mitochondrial DNA; mtROS, mitochondrial reactive oxygen species; ASC, apoptosis-associated speck-like protein containing a CARD; NEK7, NIMA-related kinase 7; NLRP3, NOD-like receptor family pyrin domain-containing 3; UPS, ubiquitin-proteasome system.

Genetics and neuropathology research confirms that misfolded α-syn is linked to PD and other neurodegenerative disorders.^92^^,^^93^ α-Syn is a neuron protein mostly found at nerve endings. In PD, α-syn is not just a bad protein inside neurons. It can also be seen as a danger signal (DAMP) by microglia cells. This triggers and worsens NLRP3 inflammasome-driven brain inflammation through connected ways, creating a vicious cycle. Outside cells, α-syn acts as a DAMP and talks to receptors on microglia. First, α-syn binds to the CD36 receptor. This brings in Fyn kinase from inside the cells.^94^ Fyn kinase then turns on NF-κB via PKC-δ.^95^ This “primes” or prepares the NLRP3 inflammasome. Second, α-syn clumps (oligomers) can turn on TLRs. This also uses NF-κB to make inflammatory signals and more NLRP3, speeding up PD.^96^^,^^97^ In microglia, these events can both prime and activate the NLRP3 inflammasome at once.^98^ Also, TLR2 facilitates the phagocytic clearance of α-syn by microglia.^99^ Following internalization, α-syn forms intracellular aggregates. These aggregates subsequently impair lysosomal function, leading to the leakage of contents such as cathepsin B into the cytosol. This directly starts NLRP3 assembly. At the same time, α-syn clumping is tied to problems in the cells’ cleanup systems. Dysfunction in the ubiquitin-proteasome system (UPS) and the autophagy-lysosome pathway contributes to the increased aggregation of α-syn.^100^^,^^101^ Fixing these systems helps clear α-syn clumps and lessens PD damage.^102^^,^^103^ This shows that inflammation from α-syn and poor protein cleanup cause each other. Inflammation harms organelles like lysosomes. Bad organelles then cannot clear α-syn well, leading to its increased buildup. This is a positive feedback loop. Also, active NLRP3 inflammasomes make ASC specks. These specks themselves can cause increased inflammation. In PD mouse brains stressed by α-syn fibers, ASC specks can trigger more NLRP3 activation.^104^ This is another way the inflammation signal boosts itself. In summary, in PD, α-syn does not use separate, straight-line pathways. It uses a connected network to drive NLRP3 activation. It works as a DAMP to turn on surface receptor signals. It also wrecks lysosome function and disrupts protein cleanup systems like UPS and autophagy. Together, this makes a cell environment where NLRP3 stays active. The NLRP3 inflammasome is activated in microglia and neurons. These mechanisms work together and make each other stronger. They form the core reason for lasting brain inflammation and steady neuron loss in PD (Figure 2).

### Mitochondrial disorders regulated by NLRP3 inflammasome activation

**Figure 3:**
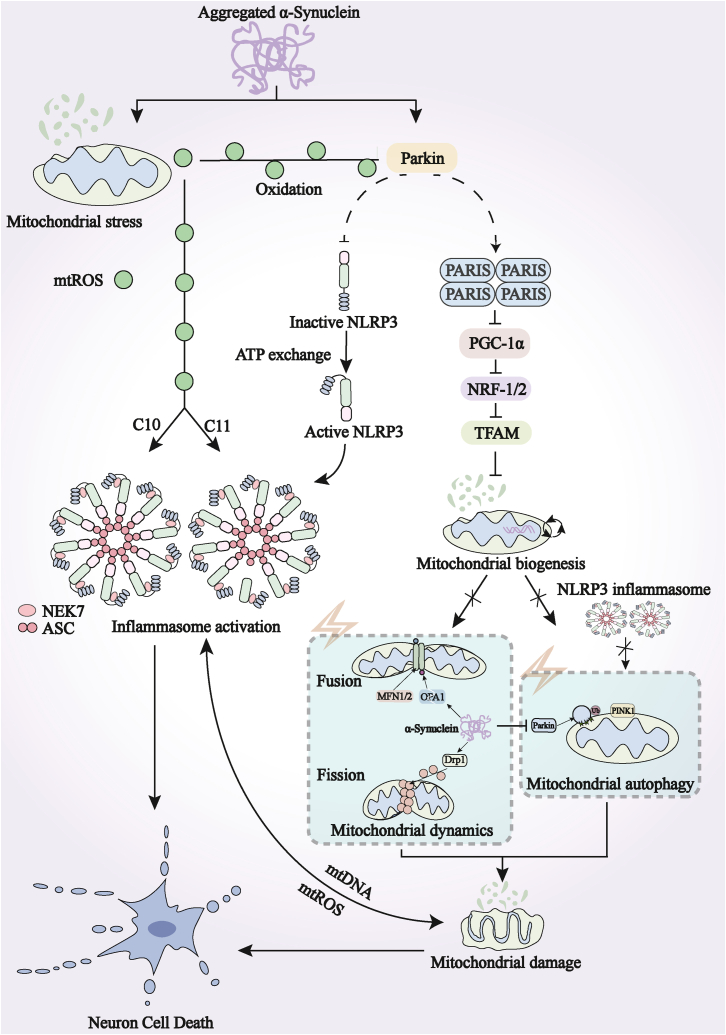
Mitochondrial dysfunction mediates α-syn-induced NLRP3 inflammasome activation and neurodegeneration Normally, Parkin inhibits NLRP3 inflammasome priming by ubiquitinating and targeting NLRP3. The mitochondria undergo a stress response in response to α-syn aggregation to produce mtROS, while the loss of Parkin protein inhibits ubiquitination degradations of NLRP3 and PARIS to inhibit NLRP3 inflammasome priming and mitochondrial biogenesis, respectively. In the context of PD, impaired mitochondrial biogenesis mediated by PGC-1α, together with defective autophagic clearance, disrupts mitochondrial quality control. This leads to the accumulation of damaged mitochondria, creating a vicious cycle of oxidative stress and energy crisis that ultimately drives neuronal dysfunction. Created with Adobe Illustrator 2025. PGC-1α, peroxisome proliferator-activated receptor γ coactivator-1α.

Mitochondrial damage and dysfunction are universally recognized as important factors in the pathogenesis of PD and other neurodegenerative diseases. These factors can lead to neuronal energy depletion, oxidative stress, and disruption of calcium homeostasis, further exacerbating neuronal death.^105^ In the pathological process of PD, aberrant and excessive deposition of α-syn can trigger mitochondrial stress and damage, leading to the release of mtROS and mitochondrial DNA (mtDNA) into the cytoplasm. These released mitochondrial components act as DAMPs, which are capable of activating the NLRP3 inflammasome. Consequently, this promotes the maturation and release of the inflammatory cytokines IL-1β and IL-18.^58^^,^^106^ The activated inflammatory signaling further exacerbates mitochondrial dysfunction and impairs the process of mitophagy.^107^^,^^108^ This leads to the subsequent release of additional mitochondrial contents into the cytoplasm, thereby further driving neuroinflammatory responses and cellular damage (Figure 3).^109^

Recent studies elucidate a complex interplay of autophagy and the NLRP3 inflammasome in PD, where autophagy acts as a critical suppressor of neuroinflammation by targeting NLRP3 degradation. Mechanistically, autophagic pathways, including chaperone-mediated autophagy (CMA) and macroautophagy, directly degrade NLRP3,^110^^,^^111^^,^^112^ while transcription factors like TFEB modulate this process by enhancing lysosomal function and autophagic flux.^32^ PTMs, such as ubiquitination and acetylation, further refine this regulatory network—DJ-1 stabilizes NLRP3 by blocking its autophagic clearance,^113^ whereas p300 degradation via CMA suppresses NF-κB-driven NLRP3 transcription.^114^ Notably, astrocytic CB2 receptor activation induces autophagy to degrade NLRP3,^115^ whereas microglial autophagy deficiency exacerbates NLRP3-mediated neuroinflammation through ROS-NLRP3 signaling.^116^ Clinically, natural compounds like kaempferol and irisin alleviate PD pathology by promoting autophagy-dependent NLRP3 degradation,^110^^,^^111^^,^^112^ alongside small molecules such as CAG and PPX that target the scribble/NADPH oxidase or dopamine receptor pathways to suppress inflammasome activation.^117^^,^^118^ However, a recent study challenges this paradigm by demonstrating that α-syn oligomers upregulate Atg5, paradoxically enhancing NLRP3 inflammasome assembly in astrocytes,^119^ suggesting context-dependent roles for autophagy proteins in inflammasome regulation. This discrepancy highlights the need to reconcile conflicting mechanisms and underscores the complexity of targeting autophagy-NLRP3 crosstalk in PD therapeutics.

Interestingly, mtROS act as an upstream signal for NLRP3 inflammasome activation, while the release of mtDNA is dependent on both NLRP3 inflammasome function and mtROS. Moreover, mitophagy not only regulates NLRP3 activation but also acts as a downstream process following NLRP3 inflammasome activation.^108^ Additionally, cardiolipin, a mitochondria-specific phospholipid located in the inner mitochondrial membrane, can induce the assembly of the NLRP3 inflammasome upon translocation to the outer membrane, further supporting the connection between mitochondrial damage and NLRP3 inflammasome activation.^109^ In microglia, mitochondrial injury triggers NLRP3 inflammasome and amplifies NLRP3 inflammasome-mediated DA neurodegeneration.^120^ Both the activation of the NLRP3 inflammasome and mitochondrial disorders play crucial roles in sustaining persistent neuroinflammation and neuronal cell death. The signaling interactions between these two factors are a *pas de deux* in PD.

### NLRP3 inflammasomes in PD and their regulation by Parkin

Parkin, the most prevalent recessive gene implicated in PD, encodes the Parkin protein, which plays a crucial role in regulating oxidative stress in DA neurons and enhancing DA neurotransmission.^121^^,^^122^^,^^123^ Parkin is an E3 ubiquitin-protein ligase located in the cytoplasmic matrix, responsible for transducing signals or degrading proteins through ubiquitination. This mechanism is beneficial in maintaining dynamic biological homeostasis. Thus, mutations in the Parkin gene can result in abnormal protein aggregation, most notably represented by α-syn.^124^^,^^125^

Misfolded α-syn impairs Parkin function, leading to disruption of the protein degradation pathways and increased cellular vulnerability to stress.^126^ Under physiological conditions, Parkin suppresses the levels of the intracellular protein PARIS, which is a Parkin-interacting substrate and a known risk factor for PD, by targeting it for degradation via the UPS,^127^ thereby promoting neuronal survival. Aberrant accumulation of PARIS contributes to PD pathogenesis. Inactivation of Parkin attenuates the clearance of downstream PARIS, resulting in its significant accumulation.^128^

Recent studies have further elucidated the pathophysiological relevance of PARIS accumulation in the context of PINK1 deficiency. In human DA neurons lacking PINK1, mitochondrial deficits are primarily attributable to impaired mitochondrial biogenesis resulting from PARIS accumulation and subsequent repression of peroxisome proliferator-activated receptor γ coactivator-1α (PGC-1α), rather than solely to defects in mitophagy.^129^ Notably, CRISPR-Cas9-mediated knockdown of PARIS in this context completely restores mitochondrial biogenesis and function without affecting mitophagy deficits, underscoring the central role of the PARIS-PGC-1α axis in PINK1-related pathogenesis. Furthermore, PINK1/Parkin-mediated regulation of PARIS directly influences DA neuron survival through modulation of PGC-1α-dependent mitochondrial biogenesis, reinforcing the concept that maintaining mitochondrial mass is as critical as clearing damaged organelles.^130^

PARIS, in turn, represses the transcription of PGC-1α by binding to the insulin response sequence in the promoter of the PGC-1α gene.^127^ PGC-1α is a master transcriptional regulator of mitochondrial function and biogenesis. Its reduction by PARIS leads to the inhibition of its downstream effectors, nuclear respiratory factors 1 and 2 (NRF1 and NRF2), and mitochondrial transcription factor A (Tfam).^131^ Tfam is a crucial transcription factor for mtDNA transcription/replication and the expression of nuclear-encoded mitochondrial proteins.^132^^,^^133^ Consequently, the suppression of Tfam activity disrupts mitochondrial biogenesis and transcription.^134^ This transcriptional impairment affects mitochondrial dynamics, leading to excessive fragmentation and compromised mitophagy. The resulting mitochondrial damage causes the release of mtDNA and mtROS. These released molecules ultimately trigger the assembly and activation of the NLRP3 inflammasome.^135^^,^^136^

The pathological consequences of PARIS-mediated PGC-1α repression have been further characterized in a conditional transgenic mouse model with DA neuron-specific PARIS expression.^137^ These mice recapitulate key PD pathologies, including progressive and selective DA neuron degeneration, striatal dopamine deficits, neuroinflammation, and motor impairments responsive to L-DOPA treatment. Single-nucleus transcriptomic analysis confirmed repression of PGC-1α and multiple mitochondria-related target genes specifically in DA neurons, providing unbiased molecular evidence for the central role of this axis in PD pathogenesis. Notably, pharmacological inhibition of c-Abl activity in this model largely prevents PD-associated pathological features, suggesting that targeting upstream regulators of PARIS may represent a viable therapeutic strategy.

Notably, in addition to the well-established role of PARIS in repressing PGC-1α, recent proximity proteomic analyses have revealed a direct mechanistic link between PARIS and the NRF2-mediated anti-oxidant defense pathway. Specifically, PARIS overexpression in PD models was found to suppress NRF2-driven neuroprotection by interfering with NRF2 nuclear translocation and transcriptional activity, independent of its effects on PGC-1α.^138^ This PARIS-NRF2 interaction represents a parallel arm through which Parkin loss exacerbates oxidative stress and neuronal vulnerability. By simultaneously impairing mitochondrial biogenesis through the suppression of PGC-1α and compromising anti-oxidant capacity via the disruption of NRF2, the accumulation of PARIS creates a dual pathogenic insult. This synergistic effect promotes the activation of the NLRP3 inflammasome and drives the degeneration of DA neurons.

Other studies have also confirmed a close relationship between Parkin and the NLRP3 inflammasome. Dysfunction of Parkin leads to the accumulation of mtROS, a major trigger of NLRP3 inflammation, generated by damaged mitochondria.^139^^,^^140^ The latest research published in *Neuron* reported the relationship between neuronal Parkin and NLRP3 inflammasome.^141^ In brief, this study emphasizes the significant role of the E3 ubiquitin ligase Parkin in suppressing the initiation and activation of NLRP3 inflammasomes in DA neurons. On the one hand, Parkin binds to NLRP3 and promotes its polyubiquitination, targeting it for proteasomal clearance. On the other hand, the accumulation of PARIS induces mtROS production, thereby facilitating the activation of NLRP3 inflammasomes and neurodegeneration in PD. Furthermore, another study found that Parkin regulates microglial NLRP3 inflammasome activation through polyubiquitination and represses neurodegeneration in PD.^76^ Together, strategies aimed at targeting Parkin-mediated microglial and neuronal NLRP3 inflammasome activity hold promise as a disease-modifying therapy for PD.

In DA neurons, Parkin-mediated inhibition of the NLRP3 inflammasome is clearly cell autonomous. The core mechanism lies in Parkin’s role as an E3 ubiquitin ligase, which directly recognizes and binds to NLRP3 within neurons, targeting it for proteasomal degradation via K48-linked polyubiquitination. This action suppresses the “priming” signal of the inflammasome at its source. Loss of this function directly leads to the accumulation of NLRP3 in neurons, activation of caspase-1, and, ultimately, cell-autonomous death.^141^ In contrast, the Parkin-NLRP3 axis in microglia primarily exerts a non-cell-autonomous immunomodulatory function. Studies show that Parkin in microglia can also promote NLRP3 degradation through K48-linked polyubiquitination. Loss of Parkin function in microglia leads to excessive activation of the NLRP3 inflammasome, resulting in the release of large amounts of inflammatory factors that exacerbate toxicity to surrounding neurons.^76^ Furthermore, evidence suggests that Parkin regulation in microglia may be more complex and that, in addition to direct ubiquitination, it may also indirectly inhibit the NLRP3 inflammasome pathway by maintaining negative feedback regulators such as the A20 protein.^142^ This cell type-specific regulatory difference, in which the focus in neurons is on maintaining intrinsic homeostasis, while that in microglia is on modulating the inflammatory environment, highlights the importance of precisely distinguishing cellular contexts in PD pathology.

The discussion above provides an in-depth elucidation of the central regulatory role of the α-syn-mitochondrial-NLRP3 axis in PD: excessively deposited α-syn inhibits Parkin-mediated ubiquitination and degradation of PARIS, leading to transcriptional repression of PGC-1α, which, in turn, promotes the activation of the NLRP3 inflammasome. Furthermore, α-syn-induced mitochondrial dysfunction and damage amplify neuroinflammation via the NLRP3 pathway. This key molecular axis not only explains the persistence of chronic inflammation in PD but also identifies precise molecular targets for intervention. Inhibiting NLRP3 inflammasome activation may block the vicious cycle that progresses from α-syn pathology to NLRP3-driven neuroinflammation and ultimately to neuronal death, thereby representing an effective therapeutic strategy against PD.

## Multi-node intervention strategies targeting the NLRP3 inflammasome

Building on a deep understanding of the pathological cascade, therapeutic strategies can be broadly classified into three directions based on their key points of intervention: from clearing the initial triggers of the disease, to inhibiting the core hub of inflammation, and further to precisely modulating the activity switches of that hub.

### PNPs inhibit α-syn aggregation and priming signals

Given that misfolded and aggregated α-syn acts as the initial trigger that sets off the downstream NLRP3-mediated inflammatory and neurodegenerative cascade, the most direct therapeutic strategy is to target this source directly. By reducing its production, inhibiting its aggregation, promoting its clearance, or blocking its intercellular transmission, we can attenuate the upstream signals that activate the NLRP3 inflammasome, thereby intervening before the inflammatory storm takes hold.

Among the current strategies for treating PD by targeting α-syn, most studies have focused on RNA interference (RNAi) technology, which involves gene silencing mechanisms targeting the α-syn mRNA level. One study demonstrated that siRNA targeting α-syn reduced endogenous α-syn expression after 2 weeks of infusion into the mouse hippocampus,^143^ indicating a favorable inhibitory effect. However, the application of RNAi in PD still requires further research and improvement because the application of viral vectors leads to the degeneration of the nigrostriatal system and neurotoxicity.^144^ The compound NPT100-18A interacts with the structural domain of the C terminus of α-syn, reduces the formation of α-syn oligomers, and ameliorates motor deficits in α-syn transgenic mice.^145^ NPT 088 has also shown good efficacy in targeting multiple misfolded proteins simultaneously, reducing α-syn aggregation and protecting nigrostriatal neurons in α-syn-overexpressing mice.^146^ Furthermore, current studies have shown that α-syn aggregation can also be regulated through the PTM mechanisms of α-syn, which includes various modification pathways such as phosphorylation (pS129),^147^ glycosylation (O-GlcNAc),^148^ and acetylation,^149^ resulting in changes in α-Syn structure and inhibiting its transport and binding.

α-Syn recognizes and binds to TLR and CD36 receptors, leading to NF-κB activation and initiation and activation of the NLRP3 inflammasome. Therefore, targeting TLR and CD36 receptors can also inhibit NLRP3 inflammasome activation, reduce the production and release of downstream inflammatory factors, and alleviate the pathological severity of PD. Additionally, regulating intracellular protein degradation function to remove α-syn can also inhibit NLRP3 inflammasome activation. Overexpression of beclin 1 has been demonstrated to inhibit NLRP3 activation by enhancing autophagy and reducing α-syn accumulation.^150^ The mTOR signaling pathway also plays a crucial role in cellular autophagy regulation.^151^ Mitoglitazone (MSDC 0160) was reported to inhibit mTOR and restore the function of autophagy,^152^ protecting DA midbrain neurons in a “chronic” genetic mouse model of PD.^153^ This suggests that modulation of mTOR can promote phagocytosis and degradation of α-syn, improving PD pathology. Except for autophagy, UPS is involved in the clearance of misfolded and aggregated proteins. Lycorine has been shown to activate the UPS and promote α-syn degradation.^154^ However, whether UPS and autophagy have a priority and mutually regulate each other in α-syn clearance remain unclear. Further research is needed to elucidate the roles of UPS and autophagy in the development of related drugs.

In addition to the aforementioned strategies, a growing body of evidence indicates that multiple plant-derived natural small molecules can directly interfere with the aggregation process of α-syn, thereby attenuating its effect on activation of the NLRP3 inflammasome at the source. These molecules act via several key mechanisms, including directly binding to α-syn monomers or oligomers to block fibril assembly, exemplified by curcumin^155^ and baicalein^156^; stabilizing the non-toxic conformation of α-syn to promote a non-aggregated state, as seen with hesperetin^157^ and piceatannol^158^; and accelerating the disaggregation of existing fibrils, demonstrated by resveratrol^159^ and (−)-epigallocatechin-3-gallate^160^ (Table 1).

**Table 1:** NLRP3 inflammasome inhibitors in preclinical studies for PD

Chemical compound	Research models	Targets	Mechanism of action	BBB permeability	Structural formula	Reference
JAC4	Rot-treated C57BL/6 mice	JWA	NLRP3, and caspase-1	unknown		Zou et al.^161^
MCC950	MPTP-treated C57BL/6 mice	NLRP3	caspase-1, ASC, and IL-1β	yes^162^		Huang et al.^22^
Talniflumate	MPTP-treated C57BL/6 mice	ASCT2	NLRP3, caspase-1, IL-1β, pro-caspase-1, and pro-IL-1β	unknown		Liu et al.^163^
KPT-8602	MPTP-treated C57BL/6 mice BV2 microglia and iBMDMs stimulated by LPS	IκBα	NLRP3, IL-1β, caspase-1, pro-caspase-1, and pro-IL-1β	unknown		Liu et al.^164^
β-Hydroxybutyrate	MPTP-treated C57BL/6 mice; BV2 cells stimulated by MPP	STAT3	NLRP3, IL-1β, and IL-18	yes^165^		Jiang et al.^166^
OLT1177	MPTP-treated C57BL/6J mice; microglia stimulated by GA	NLRP3	IL-1β and IL-18	yes^167^		Amo-Aparicio et al.^167^
Itaconate	MPTP-treated C57BL/6 mice; SH-SY5Y cells stimulated by MPP	unknown	NLRP3, IL-1β, caspase-1, and ASC	unknown		Kong et al.^168^
Glibenclamide	paraquat- and maneb-treated C57BL/6 mice; microglial cells stimulated by paraquat and maneb	ATP-sensitive potassium channels	NLRP3, pro-caspase-1, and IL-1β	unknown		Qiu et al.^169^
Pramipexole	LPS-treated C57BL/6 mice; primary mice astrocytes stimulated by LPS+ATP	Drd3	NLRP3, pro-caspase-1, pro-IL-1β, IL-1β, and ASC	yes^170^		Dong et al.^118^
Tubastatin A	6-OHDA-treated C57BL/6 mice; SH-SY5Y cells stimulated by 6-OHDA	HDAC6	NLRP3, caspase-1, IL-1β, and IL-18	unknown		Yan et al.^171^
DDO-7263	MPTP-treated C57BL/6 mice; H_2_O_2_ stimulated by PC12 cells; THP-Ms cells stimulated by ATP+LPS	Nrf2	NLRP3, caspase-1, and IL-1β	yes^172^		Xu et al.^172^
Ciprofol	MPTP-treated C57BL/6 mice; BV2 microglia stimulated by LPS	unknown	NLRP3, pro-caspase-1, and caspase-1	unknown		Wang et al.^173^
NT-0796	PD patients	NLRP3	NLRP3	unknown		Clarke et al.^174^
Rebamipide	MPTP-treated C57BL/6 mice; BV2 microglia stimulated by α-syn/MPP	NLRP3	IL-1β and IL-18	unknown		Lim et al.^175^
NT-0527	LPS+ATP-treated SD rats; PBMC stimulated by LPS+ATP	NLRP3	IL-1β	yes^176^		Harrison et al.^176^
Baicalein	rotenone-treated SD rats; SH-SY5Y cells stimulated by rotenone	CREB	p-p38, SIRT1, *p*-ERK1/2, and *p*-AMPK	yes^177^		Zhang et al.^178^
	α-syn aggregation assay	α-syn	combining with α-syn to form the Schiff base, inhibit α-syn fibril formation			Zhu et al.^156^
Hederagenin	SH-SY5Y cells stimulated by 6-OHDA	unknown	ROS	unknown		Li et al.^179^
Celastrol	MPTP-treated C57BL/6 mice	Nrf2	NLRP3, ASC, caspase-1, and IL-1β	yes^180^		Zhang et al.^181^
Urolithin A	MPTP-treated C57BL/6 mice; BV2 microglia stimulated by LPS	unknown	NLRP3, caspase-1, pro-IL-1β, and IL-1β	yes^182^		Qiu et al.^183^
Kaempferol	6-OHDA-treated SD mice; BV2 cells stimulated by LPS	p38 MAPK	NLRP3, ASC, caspase-1, IL-1β, and IL-18	yes^184^		Cai et al.^185^
Salidroside	MPTP-treated C57BL/6J mice and C57BL/10ScNJ; PC-12 cells stimulated by MPTP	unknown	NLRP3, N-GSDMD, ASC IL-18, and IL-1β	yes^186^		Zhang et al.^187^
Mangiferin	MPTP-treated C57BL/6J mice	Drp1	PINK1, Parkin, BNIP3, NIX, and FUNDC1	unknown		Wang et al.^188^
Auraptene	MPTP-treated C57BL/6J mice SN4741 cells stimulated by rotenone	Nrf2	ROS	unknown		Jang et al.^189^
Mogroside V	rotenone-treated C57BL/6J mice; SH-SY5Y cells stimulated by rotenone	caspase-3	ROS	unknown		Luo et al.^190^
Ginsenoside Rd	SH-SY5Y cells stimulated by MPP	mitochondrial respiratory complex I	ROS and MDA	yes^191^		Liu et al.^192^
Acteoside	SAL-treated C57BL/6 mice; SH-SY5Y cells stimulated by SAL	unknown	NLRP3, ASC, caspase-1, GSDMD, IL-18, and IL-1β	yes^193^		Wang et al.^194^
	MPTP-treated C57BL/6J mice; SH-SY5Y cells stimulated by MPP	Nrf2	PINK1 and Parkin			Han et al.^195^
Hyperoside	MPTP-treated C57BL/6 mice	PACAP	NLRP3, ASC, and IL-1β	unknown		Wang et al.^196^
Andrographolide	MPTP-treated C57BL/6J mice; N9 microglia stimulated by LPS+ATP+MPP	Parkin	NLRP3, IL-1β, pro-caspase-1, pro-IL-1β, and SQSTM/p62	yes^197^		Ahmed et al.^198^
Tenuigenin	MPTP-treated C57BL/6J mice; LPS-treated C57BL/6J mice; BV2 microglia stimulated by LPS	unknown	NLRP3, IL-1β, caspase-1, and pro-IL-1β	yes^199^		Fan et al.^199^
Berberine	MPTP-treated C57BL/6J mice; BV2 cells stimulated by MPP	unknown	NLRP3, ASC, caspase-1, and IL-1β	unknown		Huang et al.^200^
Echinacoside	MPTP-treated C57BL/6 mice; BV2 microglia stimulated by α-syn/MPP	TLR2/α-syn	NLRP3, ASC, caspase-1, and IL-1β	unknown		Yang et al.^201^
Baohuoside I	LPS-treated GPER C57BL/6 mice; BV2 cells stimulated by LPS	GPER	NLRP3, IL-1β, caspase-1, and ASC	unknown		Gu et al.^202^
Safranal	MPTP-treated C57BL mice	unknown	NLRP3, IL-1β, and caspase-1	unknown		Yang et al.^203^
Genkwanin	SH-SY5Y cells stimulated by MPP	unknown	NLRP3 and IL-1β	unknown		Li et al.^204^
Perillyl alcohol	MPTP-treated C57BL/6 mice; primary mice astrocytes stimulated by LPS+H_2_O_2_	unknown	NLRP3, caspase-1, IL-1β, and IL-18	unknown		Ahmed et al.^205^
Curcumin	rotenone-treated C57BL/6J mice	HDAC6	2NLRP3, ASC, caspase-1, IL-1β, and IL-18	unknown		Cai et al.^206^
	α-syn aggregation assay	α-syn	binds to α-syn monomers, prevents aggregation, and increases the reconfiguration rate			Ahmad and Lapidus^155^
Palmatine	MPTP-treated C57BL/6 mice; primary neurons or BV2 cells stimulated by MPP^+^/LPS + MPP^+^	unknown	NLRP3, caspase-1, and IL-1β	yes^207^		Zhao et al.^208^
Stigmasterol	6-OHDA-treated Sprague-Dawley rats	unknown	NLRP3 and IL-1β	yes^209^		Wankhede et al.^210^
Coixol	MPTP-treated C57BL/6 mice; BV2 cells stimulated by LPS; SN4741 cells stimulated by MPP+	unknown	NLRP3, ASC, caspase-1, IL-1β, and GSDMD	unknown		Yang et al.^211^
Erianin	MPTP-treated C57BL/6 mice; BV2 cells stimulated by LPS	unknown	NLRP3, ASC, NEK7, caspase-1, IL-1β, and IL-18	unknown		Yan et al.^212^
Inosine	LPS-treated C57BL/6 mice; BV2 cells stimulated by LPS	adenosine receptors	NLRP3, caspase-1, and IL-1β	unknown		Khanal et al.^213^
Cycloastragenol	AAV2/9-hSyn-SNCA (A53T)-α-syn-WPRE injected C57BL/6 mice; primary microglial stimulated by α-syn	unknown	NLRP3, caspase-1, and GSDMD	unknown		Feng et al.^117^
Resveratrol	modeling experiments utilizing hydrogen/deuterium exchange mass spectrometry	α-syn	remodeling of α-syn aggregation	no^214^		Illes-Toth et al.^159^
(−)-Epigallocatechin-3-gallate	α-syn aggregation assay	α-syn	dose-dependently inhibits α-syn aggregation; reduces the α-syn oligomer/fiber volume	yes^215^		Jha et al.^160^
Piceatannol	α-syn aggregation assay PC12 cells	α-syn	inhibits the formation of α-syn fibrils and destabilizes preformed filament	unknown		Temsamani et al.^158^
Hesperetin	α-syn aggregation assay; PC12 cells and *C. elegans* NL5901 stimulated by α-syn	α-syn	interferes with α-syn initial nucleation and slows the elongation rate, inhibits α-syn fibril formation	yes^216^		Wang et al.^157^
Tectorigenin	α-syn and tectorigenin aggregation model	α-syn	binds to α-syn, leading to microenvironmental changes in the tertiary structure of α-syn	unknown		Tu et al.^217^
Dihydromyricetin and Salvianolic acid B	α-syn-transfected H4 cells; homozygous transgenic mice expressing WT-α-syn	α-syn	degrade α-syn aggregates	unknown		Wu et al.^218^

These natural small molecules not only provide chemical tools for inhibiting the pathological aggregation of α-syn but also, owing to their frequent origins in dietary or traditional medicinal plants, often exhibit favorable biocompatibility and multi-target regulatory potential. They, thus, represent a rich resource of candidates for the development of PD therapeutic strategies that combine anti-aggregation and anti-neuroinflammatory functions.

### PNPs ameliorate mitochondrial dysfunction and oxidative stress

Mitochondrial dysfunction and oxidative stress serve as pivotal upstream events driving NLRP3 inflammasome activation in PD. Pathological aggregation of α-syn, loss of Parkin function, and other insults can impair mitochondria, leading to a burst of mtROS, a collapse in energy metabolism, and the release of DAMPs such as mtDNA. These events potently activate the NLRP3 inflammasome, creating a vicious cycle of neuroinflammation and neuronal death.^219^ Owing to their multi-target nature, PNPs are capable of multi-dimensional intervention at this critical juncture. For instance, baicalein promotes mitochondrial biogenesis and improves mitochondrial function at its source by activating the CREB/PGC-1α pathway^178^; Mangiferin and andrographolide balance mitochondrial dynamics by regulating proteins like Drp1, thereby inhibiting pathological fission^188^^,^^197^; while auraptene and mogroside V, leveraging potent anti-oxidant capacity, directly scavenge mtROS and activate anti-oxidant defense pathways like Nrf2 to mitigate oxidative stress damage.^189^^,^^190^ Of particular importance, PNPs such as hederagenin, acteoside, and andrographolide can also enhance PINK1/Parkin-mediated mitophagy, effectively clearing damaged mitochondria and preventing the release of inflammatory signals.^179^^,^^195^^,^^197^ Simultaneously, components like ginsenoside improve energy metabolism by protecting the function of mitochondrial respiratory chain complexes^192^ (Table 1). These mechanisms are not isolated but operate synergistically to collectively stabilize the mitochondrial network, reduce oxidative pressure, and diminish DAMP release, thereby attenuating the “priming” and “activation” signals for the NLRP3 inflammasome at their origin. This underscores the unique advantage and potential of PNPs as a multi-node intervention strategy in rectifying the core pathological disturbance in PD—the imbalance in mitochondrial quality control.

### Targeting of the NLRP3 inflammasome

Once α-syn pathology is established, the NLRP3 inflammasome in microglia becomes the central hub and amplifier that connects upstream triggers to downstream effects. Therefore, directly targeting the assembly or activity of NLRP3 represents the most central strategy for suppressing neuroinflammation. By inhibiting NLRP3, ASC, or caspase-1, the production of key pro-inflammatory cytokines such as IL-1β and IL-18 can be effectively blocked, and pyroptosis can be reduced, thereby directly alleviating the toxic inflammatory environment for neurons. Many plant-derived compounds with anti-inflammatory properties have been confirmed to exert their critical effects precisely through this mechanism.

Targeted administration of NLRP3 inhibitors can selectively suppress its activation, thereby ameliorating neuroinflammation and the severity of pathological conditions. Directly targeting the NLRP3 inflammasome via small molecules is specific and less invasive than cytokine blockade. Several such inhibitors have been reported to prevent PD pathology in models of PD (Table 1). In addition to the preclinical inhibitors, several NLRP3-targeting compounds have advanced into clinical trials, offering promising translational avenues for PD therapy (Table 2).

**Table 2:** NLRP3-targeting compounds in clinical trials for PD

Drug	Phase	Strategic decision	ID/Source
IZD174	phase 1	withdrawn	NCT04338997
VENT-02	phase 2	terminated	NCT06822517
VTX3232	phase 2	completed	NCT06556173
OLT1177	phase 2	not yet recruiting	NCT07157735
NT-0796	phase 1	completed	Clarke et al.^174^
ISM8969	phase 1	not yet recruiting	^220^

ID: ClinicalTrials.gov ID.

MCC950, a specific inhibitor of NLRP3, directly interacts with the Walker B motif within the NACHT domain of NLRP3^221^ and has demonstrated significant inhibition of NLRP3 activity in numerous experiments,^222^^,^^223^ effectively impeding the progression of neuroinflammation. Strikingly, OLT1177 has been identified to effectively penetrate the BBB and selectively inhibit the oligomerization and activation of the NLRP3 inflammasome.^167^^,^^224^^,^^225^ This compound attenuates inflammatory responses, reduces the levels of α-syn in the brain, and provides protection to DA neurons. Additionally, the XPO1 inhibitor KPT-8602, currently undergoing clinical trials, holds promising therapeutic efficacy in both *in vitro* and *in vivo* models of PD by blocking NLRP3 priming, thereby alleviating neuroinflammation.^164^

Furthermore, natural compounds have been found to exert anti-PD effects by inhibiting the NLRP3 inflammasome. Celastrol,^181^ a potent anti-inflammatory and anti-oxidative pentacyclic triterpene, prevents the loss of DA neurons and mitigates neuroinflammation by inhibiting the expressions of NLRP3, ASC, and caspase-1 genes. Kaempferol is a natural flavonoid compound that protects DA neurons in various PD models by reducing cleaved CASP1 expression and promoting the ubiquitination and subsequent degradation of NLRP3.^110^ Andrographolide, a labdane diterpene and the main bioactive constituent found in the herb *Andrographis paniculata*, has shown the ability to suppress the activation of the NLRP3 inflammasome and rescue the loss of DA neurons.^198^

Direct NLRP3 inhibitors such as MCC950 represent a rational therapeutic approach by specifically blocking NLRP3 oligomerization, yet their high specificity may also impose limitations. PD is a multi-factorial disorder driven by interconnected pathological processes—including α-syn aggregation, mitochondrial dysfunction, and oxidative stress—that collectively prime and activate the NLRP3 inflammasome.^111^ Focusing solely on the final common pathway risks overlooking these critical upstream drivers. In this context, PNPs offer a unique and compelling strategic advantage due to their inherent multi-target and pleiotropic nature.

Activation of the NLRP3 inflammasome involves a two-signal process, revealing multiple nodes for potential intervention. With their complex phytochemical profiles, PNPs are uniquely positioned to provide more comprehensive modulation. They can simultaneously regulate several key pathways, for example, scavenging ROS to reduce priming signals, inhibiting α-syn aggregation, enhancing mitophagy to clear damaged mitochondria, and ultimately suppressing NLRP3 inflammasome assembly and subsequent pro-inflammatory cytokine release.^226^^,^^227^ This multi-node interference creates a synergistic protective network that addresses the complexity of the disease more holistically than single-target drugs.

Moreover, many PNPs confer additional pharmacological benefits crucial for neuroprotection beyond inflammasome inhibition. These include potent anti-oxidant, anti-apoptotic, and neurotrophic effects.^228^^,^^229^^,^^230^ The synergy between NLRP3 inhibition and these complementary mechanisms may translate into improved therapeutic outcomes. Finally, the long history of use of many medicinal plants in traditional systems provides an empirical basis for their neuroactivity and suggests a potentially favorable safety profile worthy of focused investigation. Thus, a review of PNPs targeting the NLRP3 inflammasome aims to highlight a valuable reservoir of multi-functional, bio-inspired candidates aligned with the multi-factorial pathogenesis of PD. Such an approach may accelerate the discovery of novel leads or combination strategies capable of effectively modifying disease progression.

### Targeting of NLRP3 PTMs

The activation of the NLRP3 inflammasome is not a simple on or off switch but is rather precisely regulated by various PTMs such as ubiquitination, phosphorylation, and nitrosylation, which function as molecular switches controlling its activity. In the chronic pathological milieu of PD, the balance of these modifications is disrupted, leading to the excessive activation of NLRP3. Therefore, targeting these PTMs represents a more refined and selective therapeutic strategy, aiming to restore normal physiological regulation of the inflammasome and avoid the risks associated with global immune suppression.

As research progresses, the importance of PTMs in the activation of the NLRP3 inflammasome is being unveiled. Targeting these mechanisms can effectively suppress the activation of NLRP3. Phosphorylation and ubiquitination are the two extensively studied types of PTMs. In terms of phosphorylation, studies have revealed that the AKT-specific agonist SC-79 can activate the AKT kinase domain to bind with the NLRP3 LRR domain and phosphorylate serine 5, thereby restricting NLRP3 oligomerization and inhibiting inflammasome assembly.^78^ Conversely, the inhibitor D4476 disrupts the binding of NEK7 to NLRP3 and subsequent NLRP3 activation by promoting dephosphorylation at site S803.^231^ Targeting phosphorylation modifications has proven effective in inhibiting NLRP3 activation.

In recent years, a growing body of research has revealed that PNPs can exert inhibitory effects by modulating PTMs of the NLRP3 inflammasome. For instance, curcumin suppresses the assembly and activation of the NLRP3 inflammasome by blocking HDAC6-mediated deacetylation at lysine 84 (K84) of the NLRP3 protein, thereby reducing its stability.^206^ Another flavonoid, kaempferol, promotes the ubiquitination of NLRP3, leading to its accelerated degradation in an autophagy-dependent manner, which effectively inhibits NLRP3 inflammasome activation.^110^ These examples demonstrate that directly intervening in the PTM status of NLRP3—particularly the balance between acetylation and ubiquitination—represents a key molecular mechanism through which PNPs regulate its function.

Collectively, PTMs represent critical signals in the activation cascade of the NLRP3 inflammasome, and targeting PTMs for inhibiting NLRP3 activation shows promise for drug development. However, recognizing different PTMs on NLRP3 may serve distinct and significant functions. Developing therapeutic strategies that specifically target PTMs requires a deep understanding of the pathophysiological mechanisms and consideration of the dual role of these modification mechanisms in both health and disease. In the field of drug development, it is imperative to explore strategies that specifically target disease-related modification pathways while preserving essential cellular functions.

## Summary and outlook

Although existing evidence supports the role of the NLRP3 inflammasome in PD pathology and the therapeutic potential of PNPs, several important confounding factors and limitations in current research warrant cautious interpretation. First, most PNP studies employ crude extracts, which are chemically complex and heterogeneous, making it difficult to identify specific active molecules, establish precise dose-response relationships, and ensure batch-to-batch consistency—all of which severely hamper standardization and drug development. Second, the heavy reliance on acute toxin-induced animal models such as MPTP fails to fully recapitulate the chronic, progressive nature and complex pathological network of human PD, and discrepancies between different models may lead to inconsistent conclusions. Furthermore, a systematic evaluation of PNPs is generally lacking, especially concerning their long-term safety, their potential off-target effects with a specific focus on systemic immune defense, and their interactions with existing PD medications. Finally, how individual variables such as age, genetic background, and gut microbiota influence PNP efficacy remains poorly addressed. While these limitations do not diminish the value of PNPs, they highlight the need for a stronger research foundation. Future studies should employ more human-relevant models, investigate the pharmacokinetics and mechanisms of purified PNP components, and systematically evaluate their safety and drug interactions.

In the pathological process of PD, the activation of the NLRP3 inflammasome represents a central event subject to multi-faceted regulation. The precise molecular mechanism by which mtROS participate in the activation of the NLRP3 inflammasome remains a critical and highly contentious scientific question in the field of neuroimmunology that is yet to be fully elucidated. Currently, two competing primary viewpoints exist within the academic community. The first posits that mtROS serve as a direct and essential triggering signal for NLRP3 activation. This hypothesis suggests that mitochondrial damage or dysfunction leads to a burst of mtROS production, which may be released into the cytosol via pathways such as the voltage-dependent anion channel (VDAC).^232^ Once in the cytosol, mtROS can induce conformational changes in specific redox-sensing proteins, enabling them to directly bind to and promote the oligomerization and activation of the NLRP3 protein.^233^ Furthermore, a body of evidence indicates that mtROS play an indispensable role in facilitating the assembly of the NLRP3-ASC complex and modulating the necessary interaction between the kinase NEK7 and NLRP3, which position it as a key effector molecule in the activation cascade.^234^^,^^235^ In contrast, the second viewpoint challenges the direct triggering role of mtROS, proposing instead that its primary function is confined to the “priming” stage of NLRP3 activation. According to this view, mtROS help create a permissive environment for subsequent inflammasome assembly rather than executing the final activation command itself. Key experimental evidence for this perspective comes from the observation that ROS scavengers effectively block the classical two-step activation protocol, a process in which cells are first primed with stimuli like LPS and then exposed to an activating signal such as ATP or nigericin. However, in cells genetically engineered to overexpress the NLRP3 protein and thereby bypass the priming requirement, the same ROS inhibitors fail to prevent inflammasome activation triggered by direct activators. An even more direct challenge comes from studies in bone marrow-derived macrophages (BMDMs), which found that multiple ROS scavengers, at concentrations sufficient to significantly lower the global cellular redox state, did not markedly affect the NLRP3 inflammasome activation process.^46^ This further undermines the status of mtROS as a necessary component of the final activation step. In summary, the exact role of mtROS in the NLRP3 pathway remains an unresolved question, namely whether it acts as the direct “spark” that ignites activation or merely as an auxiliary factor that prepares the “kindling.” The ultimate clarification of this debate awaits the application of more precise cellular models and real-time monitoring technologies.

This fundamental mechanistic uncertainty profoundly impacts our understanding of the origins of neuroinflammation in neurodegenerative diseases such as PD. Within PD’s complex pathological network, NLRP3 inflammasome activation is far from an isolated event; it is tightly interwoven with multiple programmed cell death (PCD) pathways,^236^ constituting a “multi-modal synergistic lethal network.” Mitochondrial dysfunction, a core driver of PD, produces multi-faceted and catastrophic consequences. On one hand, ATP depletion can force a shift in cell death modality from apoptosis to necroptosis,^237^^,^^238^ while the sustained opening of the mitochondrial permeability transition pore (mPTP) directly releases cytochrome *c*, initiating the classical apoptotic pathway.^239^ On the other hand, the massive mtROS leaked from the impaired electron transport chain launches simultaneous multi-pronged attacks: it can oxidize and inhibit the activity of glutathione peroxidase 4 (GPX4), a key anti-ferroptosis protein, leading to lipid peroxide accumulation and directly triggering ferroptosis^240^; it can also promote the release of mtDNA into the cytosol, activating poly(ADP-ribose) polymerase 1 (PARP1), causing NAD^+^ depletion, and apoptosis-inducing factor (AIF) nuclear translocation, resulting in parthanatos.^241^ Most importantly, these mtROS molecules and their associated mtDNA are themselves potent signals for activating the NLRP3 inflammasome.^242^ Concurrently, endoplasmic reticulum stress (ERS) induced by the accumulation of misfolded α-syn deeply engages with this network via its two core unfolded protein response (UPR) pathways, protein kinase r-like endoplasmic reticulum kinase (PERK) and inositol-requiring enzyme 1 (IRE1).^243^^,^^244^ The PERK/CHOP pathway can downregulate the anti-apoptotic protein Bcl-2, transmitting the ERS signal to the mitochondrial apoptosis pathway.^245^ Meanwhile, the IRE1 pathway can activate the transcription factor NF-κB, providing a crucial “priming” signal for the transcriptional upregulation necessary for NLRP3 inflammasome assembly.^246^ Additionally, calcium dyshomeostasis caused by ERS couples with diminished mitochondrial calcium buffering capacity, further exacerbating energy crisis and oxidative stress.^247^ Thus, in PD, the NLRP3 inflammasome plays a dual role; it acts as a key effector that converges and integrates multiple upstream signals from mitochondrial dysfunction, oxidative stress, and ERS. Simultaneously, the intense inflammatory response generated upon activation poisons neurons, damages mitochondria, and exacerbates ERS through mechanisms such as the release of IL-1β and IL-18 and the induction of pyroptosis. This forms a positive feedback loop that amplifies the entire cell death network. Consequently, future therapeutic strategies should focus on understanding and intervening in this complete, interconnected lethal network, developing combination therapies that cooperatively inhibit multiple key nodes rather than targeting a single pathway alone.

Within therapeutic strategies targeting NLRP3, PNPs have attracted significant attention due to their abundant sources and diverse anti-inflammatory activities. However, their clinical translation faces deep-seated bottlenecks stemming from mechanistic ambiguity. This makes it difficult to effectively conduct clinical trials in the context of PD based on PNPs. Synthetic small-molecule inhibitors such as MCC950 and CY-09 act through relatively well-defined mechanisms, exemplified by their direct binding to and inhibition of the NLRP3 NACHT domain. In sharp contrast, the mechanisms of action for the vast majority of PNPs remain largely unexplored and akin to a “black box.” This lack of clarity manifests at multiple levels: first, their direct molecular targets are unclear, making it difficult to determine whether they act directly on NLRP3 itself or on its upstream regulators or downstream effectors. Second, their action is often indirect; many PNPs exert powerful anti-oxidant properties that scavenge ROS, thereby potentially non-specifically weakening the “priming” signal for NLRP3 rather than directly interfering with its assembly. Third, their mechanisms are complex and diverse; plant extracts are typically multi-component mixtures that may function through synergistic or additive effects across multiple targets and pathways. This mechanistic uncertainty represents a fundamental obstacle to rational drug design, structural optimization, dosage determination, and clinical trial planning, thereby contributing significantly to the high failure rate of PNPs in translational research. Despite showing promising neuroprotective effects in various PD animal models, the vast majority of research remains at the preclinical stage, severely lacking reliable data on human pharmacokinetics, bioavailability, long-term safety, and efficacy. While their multi-component, multi-target nature might be advantageous for tackling PD’s complex pathology, it conflicts with the modern drug development principles requiring well-defined components, controlled quality, verifiable mechanisms, and clear targets. Therefore, the way to break through this bottleneck lies not in pushing crude extracts directly into the clinic but in undertaking “secondary development” by using modern pharmacological methodologies. This involves viewing PNPs as a valuable source for discovering novel lead compounds; isolating and identifying active monomers followed by systematic structural modification; or treating them as an integrated “multi-component drug system” requiring holistic study, utilizing interdisciplinary techniques like network pharmacology, proteomics, and high-throughput screening to elucidate their multi-target interaction networks. Only through such in-depth analysis and standardized research can the translational gap be bridged, unlocking the true potential of PNPs in treating NLRP3-related diseases.

However, the central therapeutic efficacy of both synthetic compounds and natural products for PD is stringently limited by the fundamental challenge of the BBB. The intricate architecture of the BBB, which comprises tight junctions, efflux transporters, and metabolic enzymes, hinders the effective entry of most large molecules, polar compounds, or efflux pump substrates into the brain parenchyma.^248^ As indicated in Table 1, the BBB permeability of many reported NLRP3 inhibitors, particularly PNPs, remains unknown, constituting a significant translational gap from preclinical research to clinical application. Therefore, enhancing brain bioavailability and optimizing target activity must be treated as equally critical core objectives in future research and development.

Current advanced strategies primarily focus on: (1) Pharmaceutical chemical modification, such as designing prodrugs to improve lipophilicity or evade efflux^249^; (2) Developing advanced delivery systems, such as nanotechnology-based carriers (liposomes or polymeric micelles) or exosomes, which can protect drugs, prolong circulation time, and achieve brain-targeted delivery via receptor-mediated transcytosis^250^; and (3) Exploring physiological methods, such as focused ultrasound for transient BBB opening.^251^ For PNPs characterized by multi-component synergy, two highly promising paths are to encapsulate the whole extract in nanocarriers for “co-delivery” and structurally optimize their core active monomers.^252^^,^^253^ In conclusion, the next generation of NLRP3-targeted PD therapies, especially those based on PNPs, must integrate “potent inhibitors” with “smart delivery systems.” Only by overcoming the BBB hurdle can these compounds, which have shown great promise in cellular and animal models, truly bring hope to PD patients.

Finally, understanding the role of the NLRP3 inflammasome in PD necessitates considering its cell type specificity within the CNS. Current evidence strongly suggests that microglia are the core engine driving NLRP3-mediated neuroinflammation.^254^^,^^255^ Activation of NLRP3 in microglia not only leads to the massive release of inflammatory cytokines like IL-1β, which directly damage neurons, but can also induce astrocytes to polarize toward a pro-inflammatory, neurotoxic phenotype, collaboratively establishing and maintaining a harmful neuroinflammatory microenvironment. In contrast, the pathological role of NLRP3 within neurons themselves is more complex and debated. Although some studies, such as those in certain pain models, suggest that neuronal NLRP3 may be involved in pathological processes, a key question in the context of PD remains: whether it is sufficient to independently assemble and directly execute the pyroptosis program, thereby actively causing neuronal death rather than merely acting as a passive victim of the inflammatory environment. This requires more definitive evidence. Resolving this cell type specificity puzzle is crucial for developing precise targeted therapies. For instance, an ideal strategy might involve specifically inhibiting NLRP3 in microglia to control neuroinflammation while avoiding interference with potentially physiologically relevant NLRP3 signaling in neurons. To this end, future research should employ high-resolution technologies, including single-cell RNA sequencing and spatial transcriptomics, to map the dynamic expression and activation profiles of NLRP3 and its related signaling across distinct cell types. This work should utilize PD patient samples or human-relevant models such as iPSC-derived brain organoids. Simultaneously, utilizing cell type-specific knockout or reporter gene animal models will allow for *in vivo* validation of the relative contribution of NLRP3 from different cellular sources to PD pathogenesis and progression. These studies will not only clarify fundamental biological controversies but also provide critical theoretical groundwork for developing the next generation of cell type-targeted, side effect-minimized NLRP3 inhibitors, ultimately propelling the birth of effective therapeutic strategies against PD and other neurodegenerative disorders.

While several excellent reviews have addressed the role of NLRP3 in neurodegenerative diseases or the anti-inflammatory properties of natural products in general, this review uniquely focuses on the convergence of these two fields. Specifically, we provide a systematic and critical analysis of PNPs as multi-target modulators of the NLRP3 inflammasome within the specific pathological context of PD. Unlike previous summaries that often treat NLRP3 inhibition or PNPs in isolation, our work delineates a multi-node intervention framework. This framework connects upstream triggers such as α-syn aggregation and mitochondrial dysfunction with the core inflammatory hub of NLRP3 assembly and activation, as well as its downstream consequences. We further compile and analyze an extensive list of PNPs (Table 1), not only cataloging their effects but also dissecting their potential mechanisms of action—from direct inflammasome inhibition to modulation of upstream priming signals and PTMs. By bridging structural biology, immunology, and translational phytopharmacology, this review aims to highlight PNPs as a strategic reservoir for developing novel, multi-target therapeutic candidates against NLRP3-driven neuroinflammation in PD, while also candidly addressing the associated challenges in mechanistic clarity and clinical translation.

## Acknowledgments

We would like to express our gratitude to Chengdu Medical College for providing all the facilities required for the completion of this review. We gratefully acknowledge the use of Adobe Illustrator 2025 for creating the schematic illustrations and KingDraw software for rendering the 2D chemical structures presented in this work. This study was financially supported by Henan Province key research and development project (231111312900) and the 10.13039/501100001809National Natural Science Foundation of China (nos. 82274612, 32301030, and 82305087).

## Author contributions

Conceptualization, S.Z., Q.P., and Y.S.; review, F.Z., J.S., B.L., Z.L., C.Z., and C.Z.; writing – original draft, H.L. and Y.S.; writing – review & editing, S.Z., Q.P., and Y.S.; supervision, S.Z., Q.P., and Y.S.; funding acquisition, Y.S.

## Declaration of interests

The authors declare that no conflicts of interest exist.
